# Child Healthcare and Immunizations in Sub-Saharan Africa During the COVID-19 Pandemic

**DOI:** 10.3389/fped.2020.00517

**Published:** 2020-08-06

**Authors:** Danilo Buonsenso, Bianca Cinicola, Memenatu Ngaima Kallon, Francesco Iodice

**Affiliations:** ^1^Department of Woman and Child Health and Public Health, Fondazione Policlinico Universitario A. Gemelli IRCCS, Rome, Italy; ^2^Istituto di Microbiologia, Università Cattolica del Sacro Cuore, Rome, Italy; ^3^Department of Pediatrics, Sapienza University of Rome, Policlinico Umberto I, Rome, Italy; ^4^Ministry of Health and Sanitation, Freetown, Sierra Leone; ^5^Institute of Neurology, Fondazione Policlinico Universitario A. Gemelli IRCCS, Rome, Italy; ^6^Department of Neuroscience and Neurorehabilitation, San Raffaele Pisana IRCCS, Rome, Italy

**Keywords:** COVID-19, Africa, SARS-CoV-2, vaccination, children, malaria, pneumonia

## Abstract

Since COVID-19 in the pediatric population is infrequently severe, the indirect costs of the pandemic, related to the measures implemented to deal with the spread of the virus, can be worse than the infection itself. To assess this issue, we evaluated the number of children vaccinated or evaluated for the most common diseases in a poor village in Sierra Leone, showing a worrisome drop in vaccinations performed and children evaluated for acute diseases. Our preliminary findings highlight that support is needed to guarantee basic services to children during the COVID-19 pandemic, particularly in poor settings where preventive measures can be lifesaving in the long term.

## Introduction

After the first description of clusters of pneumonia due to SARS-CoV-2 in China, the virus has spread all over the world. Monitoring activities and quarantine are playing an important role as rapid corrective strategies, but these processes could be very critical in the world's poorest health systems. Since COVID-19 in the pediatric population is infrequently severe (1), the indirect costs of the pandemic, related to the measures implemented to deal with the spread of the virus, can be worse than the infection itself.

In this scenario, the COVID-19-related effects on child healthcare in Africa can be massive (2), especially in the remote areas of sub-Saharan Africa, where health, economic, and social monitoring systems are poorly developed. This situation may even worsen, considering that several countries are soon entering the rainy season, which has major consequences for daily life and a potential impact on the rate of SARS-CoV-2 spread. A decrease in vaccinations could be among the most feared indirect effects of the pandemic, leading to a possible increase in morbidity and mortality in a population that has been considered to be less involved as regards the severe clinical manifestations of SARS-CoV-2 compared to adults (1).

For these reasons, we aimed to understand the potential indirect impact of COVID-19 on child vaccinations and basic healthcare in a typical poor peripheral area of Sierra Leone (Kent, Rural Western Area, Sierra Leone). This area is not provided with basic services such as electricity and running water, with people needing to go to local streams for daily provision of water (3, 4). Moreover, the area, although not far from the capital, Freetown, refers to local health centers for vaccinations as well as for routine antenatal and postnatal care. The area has an overall 90% vaccination coverage, which is in line with the overall national data published by the World Health Organization and UNICEF in 2019 (http://158.232.12.119/immunization/monitoring_surveillance/data/sle.pdf). This research is part of a project that we started in 2015 in Bureh Town, Rural Western Area, Sierra Leone, partnering with the nearby community health center of Kent (Rural Western Area, Sierra Leone), which is the referral center for basic pediatric health services, from child visits (health controls and acute diseases) to vaccinations.

In Sierra Leone, the first restrictive governmental measures (border control and closure, social distancing) were dated March 23, with total lockdown declared on April 3 (https://www.imf.org/en/Topics/imf-and-covid19/Policy-Responses-to-COVID-19#S). As in many African countries, healthcare in Sierra Leone is free only for children under 5 years and pregnant women. Moreover, since health centers cover a wide area, most people need to pay for transport services to reach health centers, which is generally difficult and is even harder after COVID-19-related drop in income. Also, quarantine and fear of contagion, as well as the fear of being recognized as a COVID-19 case and stigmatized [a known problem in Africa, as already described with HIV and Tuberculosis (4)] might impact the routine child healthcare in these settings.

## Methods

We performed a retrospective cross-sectional study collecting the number of children under 5 years of age vaccinated for the most common diseases at the Kent Community Health Post (referral from the local communities of Kent, Bureh, Checkpoint, Bonga Wharf, and Quarry; estimated population of about 5,000 people) from March 1, 2020, to April 26, 2020, and compared the results to the same period of the previous year (March 1, 2019, to April 26, 2019). In the same periods, the diagnoses of malaria, pneumonia, and diarrhea were also assessed for the two different populations of children under 5 years of age as well as immunization for tetanus in pregnant women. During the lockdown, the health center continued to provide the same activities as in the pre-lockdown era, with all services guaranteed, from acute disorders to immunization services. Therefore, there was no reduction in the health activities offered to the local population due to COVID-19 restrictions. The data were collected, as absolute numbers, by the center's health workers in accordance with the usual rules held in Sierra Leone. Cases were retrospectively obtained using the health facility routine activity reporting forms. In many peripheral Sub-Saharan Africa health centers, data are collected by the health facility on a register by crossing off with a pen the type of vaccinations performed or the disease diagnosed, without registering personal data. Peripheral health centers have a registry book with dedicated pages for registration of diagnoses and type of vaccinations. For example, they have boxes to tick labeled “PENTA1,” “PENTA2,” and “PENTA3.” This means that “PENTA1” is the first, and the others are the boosters.

Written data were subsequently transferred to an electronic database and analyzed with SPSS software v.36. A comparison between the number of vaccinated children in the two periods of time, for each type of vaccine, was performed by applying a Wilcoxon test.

The study was approved by a local commission composed of the research team of the Kent Community Health Post, the headman of the community, and the old men of the village, in a similar way as happens for all important political and economic decisions in the examined area (n24_may-2020). Personal data were not collected.

## Results

We noticed that a lower number of children received vaccination in 2020 compared with 2019, ranging from 50 to 85% depending on the individual vaccine analyzed, including BCG and OPV1, which are given directly at birth in Sierra Leone (see Table 1 for full details). Moreover, we also noticed a drop in common diagnoses, as has been happening also in developed countries (5), and a reduction of the most common clinical conditions (malaria, pneumonia, and diarrhea), although no increases in deaths were reported (Table 2). There was a 50–80% drop in vaccination in 2020 compared to the previous year (*p* < 0.0005). Conversely, although the number of common diagnoses was lower in 2020, there was not enough evidence to be sure that this is a true difference (*p* > 0.05).

**Table 1:** Vaccination performed in a community health center in Sierra Leone during COVID-19 lockdown, compared with the previous year.

**Vaccination**	**Children under 5 years of age**			
	**01/03/2019 to 26/04/2019**	**01/03/2020 to 26/04/2020**	**Change (%)**	***P*-value**
BCG	36	17	−52.7	*p* < 0.0005
OPV0	36	17	−52.7	*p* < 0.0005
OPV1	58	17	−70.7	*p* < 0.0005
PENTA1	58	17	−70.7	*p* < 0.0005
PCV1	58	17	−70.7	*p* < 0.0005
ROTA1	58	17	−70.7	*p* < 0.0005
OPV2	71	15	−78.9	*p* < 0.0005
PENTA2	71	15	−78.9	*p* < 0.0005
PCV2	71	15	−78.9	*p* < 0.0005
ROTA2	71	15	−78.9	*p* < 0.0005
IPTI1	49	15	−69.4	*p* < 0.0005
OPV3	67	15	−77.6	*p* < 0.0005
PENTA3	67	15	−77.6	*p* < 0.0005
PCV3	67	15	−77.6	*p* < 0.0005
IPTI2	44	15	−65.9	*p* < 0.0005
IPV	67	15	−77.6	*p* < 0.0005
IPTI3	45	22	−51.1	*p* < 0.0005
Measles	64	22	−65.6	*p* < 0.0005
Yellow fever	64	22	−65.6	*p* < 0.0005
Measles 2nd	49	8	−83.7	*p* < 0.0005

*BCG, Bacille Calmette Guerin; OPV, oral polio vaccine; PENTA, diphtheria, pertussis, tetanus, hepatitis B, and hemophilus; PCV, pneumococcal vaccine; ROTA, rotavirus; TT, tetanus toxoid vaccine; IPTi, intermittent preventive treatment in infants. The number associated with the abbreviation denotes initial and booster vaccinations. Data are collected as absolute numbers*.

**Table 2:** Main acute illnesses evaluated in a community health center in Sierra Leone during COVID-19 lockdown compared with the previous year.

**Diseases**	**Children under 5 years of age**			
	**01/03/2019 to 26/04/2019**	**01/03/2020 to 26/04/2020**	**Change (%)**	***P*-value**
Malaria (clinical diagnosis)	211	126	−40.3	*P* >0.05
Malaria (confirmed)	120	90	−25	*P* >0.05
Pneumonia	129	74	−42.6	*P* >0.05
Diarrhea	30	15	−50	*P* >0.05
Death	0	0	/	na

*Data were collected as absolute numbers*.

## Discussion

Although we only analyzed preliminary data from an extremely poor area, they show an important decline in the vaccination rate and, although the difference was not significant, in child visits for acute diseases in the lockdown period in a rural area of Sierra Leone. The reduction in vaccination rates is particularly evident for booster vaccinations, suggesting that parents are not bringing their children back to health facilities for the subsequent controls and vaccinations, possibly because of fear of contagion. It is important to remark also the reduction of vaccinations given at birth in Sierra Leone in our sample (BCG and OPV1). This suggests either a decrease in the number of births or an increase in deliveries at home due to a fear of being infected in health facilities, which may be a high risk for woman and child health. These data highlight the need for active surveillance of births and immunizations registered in peripheral health centers in order to better understand the meaning of our findings. No increase in deaths in the area was reported. Anecdotical reports from our team highlighted no problems regarding vaccine supplies in the area due to COVID-19 restrictions, so the reduced vaccinations are not related to vaccine supplies. Also, it is important to highlight that, during the lockdown, the health center maintained the same activities as in the pre-lockdown era, with all services guaranteed, from acute disorders to immunizations services. Therefore, there was no reduction of the health activities offered to the local population because of COVID-19 restrictions.

We are aware of the limitations of our findings. In particular, this is a retrospective study concerning a limited time period, we collected absolute numbers, and no comprehensive epidemiological data for the area are currently available (such as birth rates from local villages) since the current pandemic is creating a high workload for local workers and, at the same time, limits their possibility of interacting with other offices. However, we also understand that collecting more robust data in very remote, peripheral, rural areas of Sierra Leone is not easy, particularly in a period when movements between areas are not allowed. However, these findings are new and bear potential significant implications. Local health workers anecdotally reported they had the feeling that people could not afford routine care (from transport fees to visit fees for those over 5 years of age), and our data provide, to the best of our knowledge, the first evidence of a possible drop in access for basic but priority pediatric services in remote areas, while similar rates have already been reported in the adult population of South Africa (6). A similar scenario is currently happening in the United States, where a recent nationwide analysis of vaccine information found that measles, mumps, and rubella vaccinations had dropped by 50% during the COVID-19 outbreak (6). However, while the U.S. has tools to face this challenge, and the American Academy of Pediatrics is working hard to support pediatricians and families to ensure vaccination coverage, low-income rural/peripheral settings in Sierra Leone do not have easy access to the appropriate instruments to ensure that families bring children to health centers, and our data on a small sample population confirm what the World Health Organization predicted as one of the possible effects of the lockdown on child healthcare (7, 8). Importantly, a drop in vaccination coverage in Africa bears potentially devastating consequences for child health.

Although our data must be interpreted with caution, since we report only a single experience from an area of Sierra Leone, they clearly indicate a new scenario. It is not easy for low-income rural/peripheral settings in Sierra Leone to respond to COVID-19, giving rise to a potential risk that children could suffer from the indirect consequences of the pandemic more than in other parts of the world. Sources of income in peripheral/rural areas in Sierra Leone can be significantly impacted by the COVID-19 pandemic lockdown (9). Although testing, isolating, and quarantining are the best ways to prevent COVID-19 diffusion in Africa (10), this can lead to difficulties in providing basic services to the local population, as previously described (3). People from the area we analyzed are already experiencing limitations related to the pandemic in accessing basic needs, such as food, water, health services, and preventive strategies, and are now depending on donations for basic food (3). Highly vulnerable populations, such as younger children, often already compromised by malnutrition and comorbidities, face a greater risk of developing diseases or missing preventive opportunities (11). In these peripheral/rural areas in Sierra Leone, people need a comprehensive approach that aims to provide COVID-19 care facilities as well as social services and essential resources for more common problems, and economic support must not be directed only to epidemiologic research trials (12). Importantly, the rainy season is coming soon, and the situation will probably worsen.

In conclusion, despite the limitations mentioned, our findings highlight a possible drop in immunizations in children living in a peripheral, rural area in Sierra Leone, West Africa. Considering the potential short- and long-term impact of reduced immunization rates on child health, there is a need to actively monitor vaccination practices in low-resource settings to ensure that there is no break in service delivery.

## Data Availability Statement

The raw data supporting the conclusions of this article will be made available by the authors, without undue reservation.

## Ethics Statement

The study was approved by a local commission composed of the research team of the Kent Community Health Post, the headman of the community, and the old men of the village, in a similar way as happens for all important political and economic decisions in the examined area (n24_may-2020). Personal data were not collected.

## Author Contributions

DB, BC, and FI substantial contributions to the conception or design of the work. DB and MK were responsible for acquisition, analysis, or interpretation of data for the work. DB, BC, and FI drafted the work or revising it carefully for important intellectual content. All author gave final approval of the version to be published and agreed to be accountable for all aspects of the work in ensuring that questions related to the accuracy or integrity of any part of the work are appropriately investigated and resolved.

## Conflict of Interest

The authors declare that the research was conducted in the absence of any commercial or financial relationships that could be construed as a potential conflict of interest.
